# Tracking antibiotic resistance gene pollution from different sources using machine-learning classification

**DOI:** 10.1186/s40168-018-0480-x

**Published:** 2018-05-24

**Authors:** Li-Guan Li, Xiaole Yin, Tong Zhang

**Affiliations:** Environmental Biotechnology Laboratory, Department of Civil Engineering, The University of Hong Kong, Pokfulam Road, Hong Kong, 999077 China

**Keywords:** Antibiotic resistance gene, Source tracking, Machine learning classification, Metagenomics

## Abstract

**Background:**

Antimicrobial resistance (AMR) has been a worldwide public health concern. Current widespread AMR pollution has posed a big challenge in accurately disentangling source-sink relationship, which has been further confounded by point and non-point sources, as well as endogenous and exogenous cross-reactivity under complicated environmental conditions. Because of insufficient capability in identifying source-sink relationship within a quantitative framework, traditional antibiotic resistance gene (ARG) signatures-based source-tracking methods would hardly be a practical solution.

**Results:**

By combining broad-spectrum ARG profiling with machine-learning classification SourceTracker, here we present a novel way to address the question in the era of high-throughput sequencing. Its potential in extensive application was firstly validated by 656 global-scale samples covering diverse environmental types (e.g., human/animal gut, wastewater, soil, ocean) and broad geographical regions (e.g., China, USA, Europe, Peru). Its potential and limitations in source prediction as well as effect of parameter adjustment were then rigorously evaluated by artificial configurations with representative source proportions. When applying SourceTracker in region-specific analysis, excellent performance was achieved by ARG profiles in two sample types with obvious different source compositions, i.e., influent and effluent of wastewater treatment plant. Two environmental metagenomic datasets of anthropogenic interference gradient further supported its potential in practical application. To complement general-profile-based source tracking in distinguishing continuous gradient pollution, a few generalist and specialist indicator ARGs across ecotypes were identified in this study.

**Conclusion:**

We demonstrated for the first time that the developed source-tracking platform when coupling with proper experiment design and efficient metagenomic analysis tools will have significant implications for assessing AMR pollution. Following predicted source contribution status, risk ranking of different sources in ARG dissemination will be possible, thereby paving the way for establishing priority in mitigating ARG spread and designing effective control strategies.

**Electronic supplementary material:**

The online version of this article (10.1186/s40168-018-0480-x) contains supplementary material, which is available to authorized users.

## Background

Antimicrobial resistance (AMR) is becoming a global health crisis, threatening effectiveness of antibiotics to treat infections. At least 700,000 people die annually from drug-resistant infections [1]. The challenge will get worse if we do not act immediately to turn the tide against epidemic propagation of AMR. AMR mitigation thus is a critical health security challenge of this century, yet only limited progress has been achieved [2, 3]. Indeed, AMR has been substantially extended beyond medical settings to include relevant environmental compartments [4–7], such as soil and water. Their fate and behavior in different environments complicated the problem. In particular, point and non-point potential sources, as well as endogenous and exogenous antibiotic resistance genes (ARGs), make it difficult to disentangle the true origins [8–10]. Lack of comprehensive understanding in source-sink relationship in ARG dissemination dramatically impedes efficient AMR control.

Ever since discovering frequent ARG occurrences in human-related environments, considerable attention has been paid to identify potential sources [11–16]. For example, through combining PCR-derived detection frequency/intensity of target genes and environmental variables in a specific region, ARG distribution patterns that unambiguously distinguish putative sources of ARG pollution from a native environment have been studied in livestock farm and river basin [14–16]. Nonetheless, PCR bias and inhibition are always a concern with any PCR-based source-tracking method. In addition, specificity and sensitivity of single-marker tests vary among ARGs [15, 16]. Measuring limited number of predetermined representative ARGs and custom-tailored biomarkers is often confounded by inputs from a variety of sources. Therefore, accurately estimating the proportion of ARG contamination from source environments poses a grand challenge in AMR control.

Advances in high-throughput sequencing (HTS) have revolutionized the way to detect genes in complex environmental communities, providing a promising approach for comprehensive genetic profiling. Indeed, approximately thousands of ARGs have been identified through environmental metagenomic studies [17–19], their potential to be used as a means for identifying sources of ARG contamination however remains largely unexplored, in spite of a few comparative surveys of ARG composition among important sources [17–21]. The distinctive combinations of potential thousands of genetic markers in HTS-based metagenomic analysis might open up new avenues for source discrimination. However, an automated and statistical robust classification approach is necessary for routine application of metagenomics-based methods in real ARG monitoring. Machine-learning classification is an efficient tool in this big-data era, which uses comprehensive sequence profiling of samples from different source environments (e.g., sample-wise abundance matrix across marker gene sets) to train models to distinguish different source types. Through eliminating uninformative features, these algorithms select subsets of features from typical thousands of sequences that are most useful for source prediction [22], thereby allowing us to assess the likelihood that individual source contributes to the overall ARG composition in a sink sample. Recently developed classification methods (e.g., RandomForest [23] and SourceTracker [22]) have been successfully adopted for community-based source tracking [22, 24, 25]. In particular, the Bayesian classification tool, SourceTracker, uses Gibbs sampling to explore the joint possibility distribution of assigning all test sample sequences to the different source environments, featuring in directly inferring the mixing proportion of sources in a sink sample. SourceTracker allows sequences in a sink sample assigned to unknown sources, and it explicitly models a sink sample as a mixture of sources rather than predicts the entire sink sample from a single source. Rigorous comparison showed that SourceTracker outperformed other methods like naïve Bayes modeling and RandomForest classifier, even when disambiguation was difficult [22]. Despite lack of application in source tracking of ARG pollution, given the long-proposed strong correlation between microbial community structure and ARG profile [26–28], as well as the predictable variation of ARG overall patterns in environments along anthropogenic activity gradient [17–19, 29], SourceTracker could serve as a powerful tool in predicting putative sources of ARG contamination in a probabilistic framework.

In this study, rigorous analysis was conducted to comprehensively evaluate performance of SourceTracker in source prediction of ARG pollution. To uncover ARG profiles in diverse environments, 656 metagenomic datasets were retrieved from public databases, including four ecotypes, i.e., human feces (HF), animal feces (AF), activated sludge from wastewater treatment plant (WA), and natural environments (NT). Using well-established annotation pipeline, broad-spectrum ARG abundance profile was obtained for each sample. Through leave-one-out cross-validation, SourceTracker achieved excellent performance in source prediction for 656 samples by leveraging information embedded in ARG profiles. Furthermore, three ways were utilized to validate application of SourceTracker for samples with different anthropogenic impacts, including artificial configurations, influent and effluent of wastewater treatment plant (WWTP), as well as region-specific sediment samples with significant anthropogenic activity gradients. Besides general-profile-based source tracking, ARGs of particular interest, including generalist/specialist indicator and common/unique groups across ecotypes, were explored. Taken together, in combination of comprehensive ARG profiling with cutting-edge machine learning classification, capability of the novel platform in source tracking was well validated in this study, which may lead to fundamentally new strategies to address the current widespread ARG contamination.

## Results

### ARG overall distribution profile

**Fig. 1:**
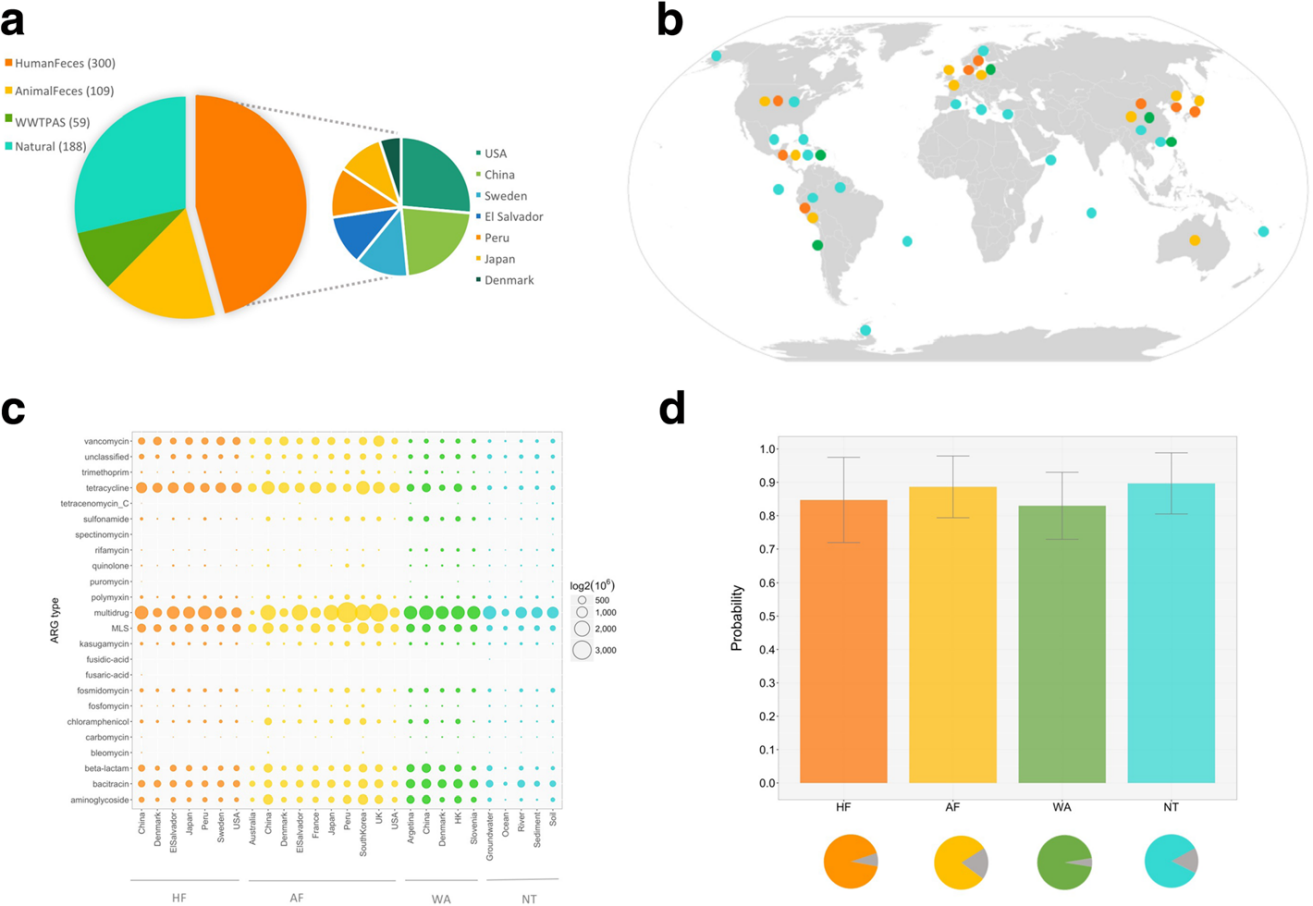
General information (including dataset composition, geographical location, and ARG abundance profile) of downloaded metagenomic datasets and preliminary validation of SourceTracker prediction performance. **a** Dataset composition among four ecotypes (i.e., HF, AF, WA, and NT) and regional distribution profile of HF samples. **b** Geographical location of global-scale metagenomic samples. **c** ARG abundance profile (log2 transformed) across 24 ARG types among different eco-subtypes. **d** Ecotype prediction by SourceTracker using leave-one-out cross-validation, in which bar chart was correctly predicted probability profile (error bar, standard deviation) among four ecotypes and pie charts were correct prediction ratio (color part) of samples in the corresponding ecotype. Consistent pairs of color-ecotype were used in all sub-figures, i.e., orange-HF, yellow-AF, green-WA, and cyan-NT

After quality check, 656 metagenomic datasets were included in this study, covering diverse environmental types (e.g., human/animal gut, WWTP, soil, sediment, ocean) and broad geographical regions (e.g., China, Japan, US, Europe, Peru) (Fig. 1a, b, Additional file 1: Table S1). Although diverse data sources were included, there is no obvious study effect observed across these dataset (Additional file 2: Figure S1). Because of uneven data sources in public databases, there were more samples of HF (*n* = 300) and NT (*n* = 188) while less samples of AF (*n* = 109) and WA (*n* = 59). Despite possible bias embedded in sample number distribution, we aim to use best of current resources to disentangle potential relationship between ARG abundance profiles of source and sink samples, especially association of environmental resistome development with anthropogenic impact. Overall, 3502 ARG reference sequences (87% out of the total 4048 reference sequences) from all 24 types in SARG database were detected in at least one of the 656 samples (i.e., 2688 ARGs were detected in HF, 2788 in AF, 2400 in WA, and 2609 in NT). The relative abundance (copies of ARG per copy of 16S rRNA gene) and richness (number of ARG types) showed obvious variability, both between and within the four ecotypes (Fig. 1c). Generally, ARGs were more abundant in AF (avg. abund. 0.78 with range 0.06~ 4.68) and HF (avg. abund. 0.52 with range 0.10~ 2.52) than WA (avg. abund. 0.37 with range 0.20~ 1.52) and NT samples (avg. abund. 0.22 with range 0~ 2.01). The top abundant ARG types differed among the four ecotypes, e.g., HF, ARGs against tetracycline, aminoglycoside, and macrolides-lincosamides-streptogramines (MLS)); AF, ARGs against tetracycline, MLS, and beta-lactam; WA, ARGs against multidrug, bacitracin, and aminoglycoside; NT, ARGs against multidrug and bacitracin. Many ARGs were widespread across ecotypes, such as tetracycline, aminoglycoside, and beta-lactam. Vancomycin-resistance genes were with low abundance in NT and WA, whereas frequently detected in both HF and AF. In addition, ARG profiles in feces samples varied by regions with different antibiotic consumption and management. For instance, much more ARGs were detected in AFs of Peru (avg. abund. = 6.40), El Salvador (avg. abund. = 6.40), and China (avg. abund. = 3.80), whereas much less detected in samples from Denmark (avg. abund. = 0.18), and antibiotic-polluted environments have the highest abundances of ARGs, such as Peru and El Salvador soil (avg. abund. = 1.20). On the contrary, much less was detected in almost pristine natural habitat, such as ocean (avg. abund. = 0.05). In agreement with previous studies [17, 18, 30], ARG abundance profiles obtained here lend evidence for the essential role of human activities in ARG development. To further investigate whether the microbial community correlated with the ARG composition, we used Procrustes analysis to correlate the two profiles. Our results showed that ARG profiles were significantly correlated to the shared bacterial compositions and structures (*P* < 0.001, based on 9999 permutations) based on Bray-Curtis dissimilarity metrics (Additional file 2: Figure S2).

### SourceTracker prediction performance

The distinct ARG abundance profile characterized by each of the four ecotypes, implied its potential in distinguishing samples with different ecotype origins. The significant correlation between ARG and community profiles revealed in this study and previous work [26–28], together with the reported successful application of SourceTracker in microbial source tracking using community profile [24, 31, 32] further lend us confidence in extending SourceTracker to broad-spectrum ARG profile-based source prediction. After five runs by leave-one-out cross-validation strategy, SourceTracker correctly predicted corresponding ecotype of 88% (578/656) samples, in particular, 92% (276/300) HF samples with predicted probability of 88%±12%, 81% (88/109) AF samples with 84%±13%, 95% (56/59) WA samples with 89%±11% and 84% (158/188) NT samples with 82%±11% (Fig. 1d). Prediction variation within five runs was observed (Additional file 3: Table S2), which might be improved by including more high-quality source samples in training datasets in future studies. Generally, pre-test by 656 samples proved applicability of SourceTracker in ARG source tracking, we then utilized three ways to further validate robustness of this method. To avoid possible bias introduced by parameter alteration (refer to the ‘Effect of parameter adjustment’ section), all SourceTracker runs of metagenomic datasets in this study were performed under default setting.

#### Artificial configuration

**Fig. 2:**
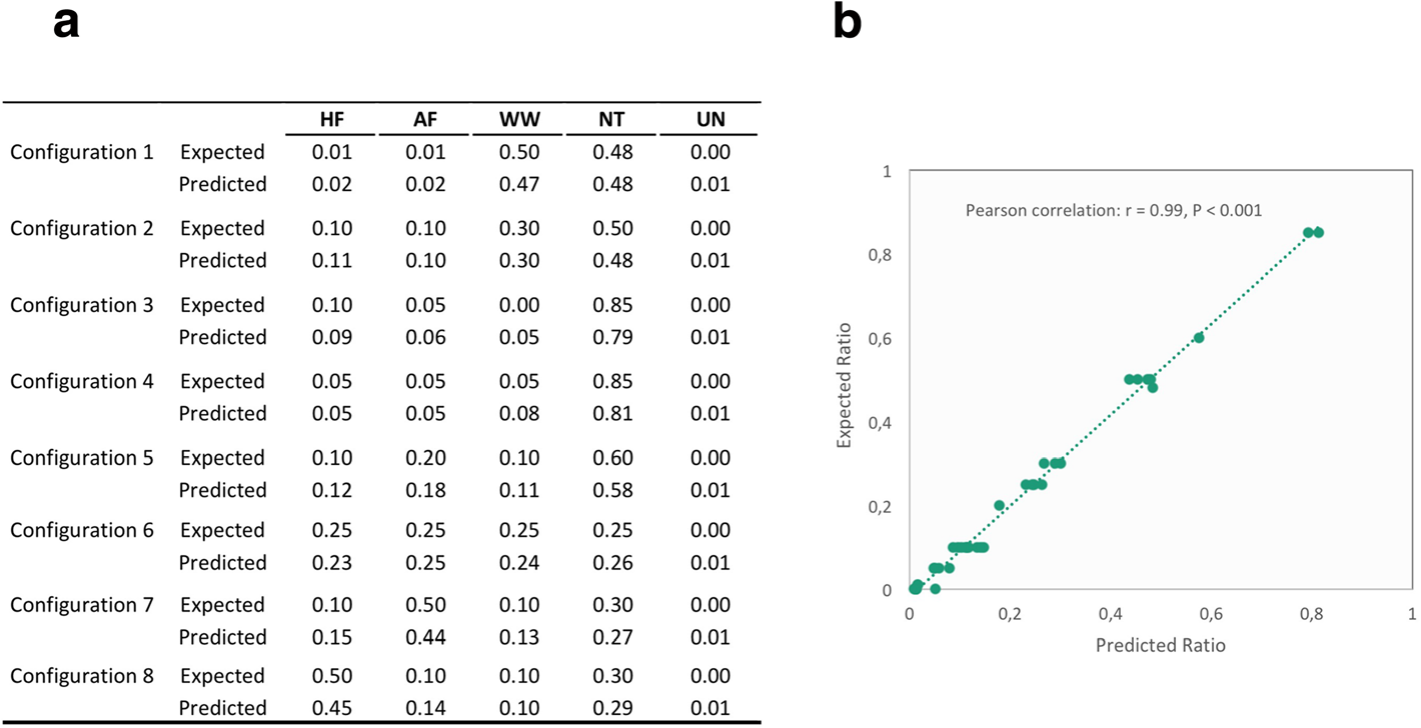
SourceTracker prediction performance validation by eight artificial configurations. **a** Predicted (result of SourceTracker prediction function) and expected (defined source proportions of four ecotypes) source proportions of the configurations. **b** Correlation analysis between the SourceTracker predicted source proportion (*X*-axis) and corresponding expected proportion (*Y*-axis)

Prior to applying SourceTracker in region-specific analysis, its potentials and limitations were firstly examined by eight artificial configurations, which were generated with different ratio of ecotype input to simulate real possible pollution levels. The averaged SourceTracker-predicted source ratio and relative standard deviation (RSD) for each configuration were presented in Fig. 2 and Additional file 3: Table S3. The results of Pearson correlation analysis demonstrated a significant correlation (*r* = 0.99, *P* < 0.001) between expected and predicted source contributions across all configurations. However, precision of the prediction appeared to be dependent on the level of contamination. This effect was clearly illustrated in Configuration 1, where low variance among runs (RSD of 8%) was observed for predicting sources with high expected ratio (47% of WA contamination), while high variance (RSD of 44%) for sources with low expected ratio (2% of AF contamination). Indeed, the similar RSD variance has been explicitly examined in application of SourceTracker in community-based source tracking [33].

#### WWTP influent and effluent

We next tested SourceTracker performance by ARG profiles in two sample types with obvious different source compositions, i.e., WWTP influent and effluent. WWTP influent is a mixture of wastewater discharged from certain communities, of which the main source is HF. Through a series of treatment processes, effluent samples are mainly from WWTP rather than HF. SourceTracker was then applied to analyze ARG profiles of four influent and effluent datasets collected at different seasons (summer or winter) (Additional file 2: Figure S3). Clearly, human/animal feces were up to 30% in influent while less than 5% in effluent and WA was predicted as the main source of the effluent samples. Because WA training datasets of activated sludge were a mixture of influent and local WWTP community, it is not surprising that certain portion of WA was also detected in the two influent samples (41% in summer and 20% in winter). Seasonal variation, as well as unknown sources were observed in the source prediction, which might be attributed to complex mixture of diverse microbial communities (e.g., bacteria groups decided by the seasonal variation of organics in the influent) and different selective pressures (e.g., antibiotics and metals) in this type of environment [34, 35].

#### Environmental samples of anthropogenic interference gradient

**Fig. 3:**
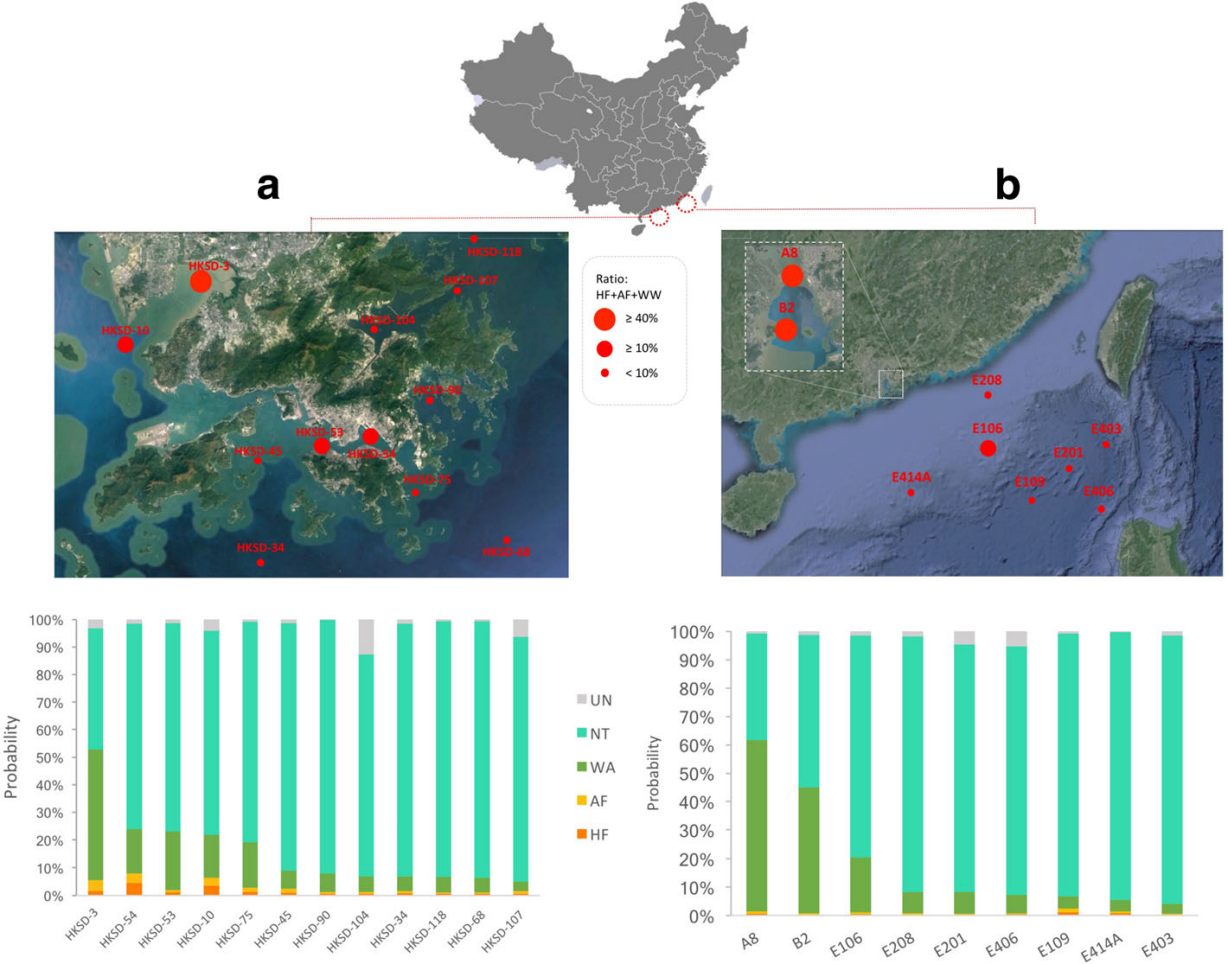
Sampling geographical location and predicted source proportion of HK sediment samples (**a**) and PRE and SCS sediment samples (**b**). In both **a** and **b**, *X*-axis were sample names and *Y*-axis were predicted source proportion (probability)

Based on above explicit evaluation, two published environmental metagenomic datasets were utilized as final test samples, i.e., two sets of region-specific samples with obvious anthropogenic activity gradient (Fig. 3, Additional file 3: Table S4).

##### Hong Kong (HK) sediment (Fig. 3a)

The results showed that 5 of 12 HK sediment samples had more than 20% feces-like contamination (i.e., aggregated proportions of feces-related sources including HF, AF, and WA). Particularly, HKSD-3 which suffered from discharges of both harbor and municipal pollutants from HK and Shenzhen areas showed a high feces-related pollution with 47% WA, 2% HF, and 4% AF. The point HKSD-10 close to HKSD-3 in outlet of harbor also showed a relatively high pollution ratio with 16% WA, 3% HF, and 3% AF. HKSD-53 and 54 sites located along a water channel that is surrounded by a high density of inhabitants showed 21 and 16% WA pollution, respectively. Close to another sewage discharge channel, HKSD-75 was also revealed 16% WA contamination. Other 7 points within areas of limited human interference were with less than 10% feces-related contamination. Overall results from SourceTracker prediction well matched regional characteristics of these sampling points.

##### Pearl River Estuary (PRE) and South China Sea (SCS) sediment (Fig. 3b)

The feces-related source contribution level to ARG profiles in sediments substantially decreased from the mouth of the Pearl River (A8) to the middle of the PRE (B2), and on to the SCS, which was in good accordance with antibiotic concentrations detected in a previous study [36]. Due to proximity to PRE region that had been heavily impacted by rapid urbanization and industrialization, significant proportion of WWTP and minor proportion of HF/AF contamination were detected at the two sampling sites, e.g., A8 of 60% WA and B2 of 44% WA. There were seven sampling locations in the SCS with varying distances to the Chinese mainland and different water depths. Except E106 collected at a location between the offshore area and the continental shelf with 20% WA, other SCS sediment samples with less than 8% WA contamination. Overall, the average pollution ratio of feces-like sources in the two PRE sediments was at least five times higher than those in the SCS sediments, reflecting distinct levels of anthropogenic interference in the two regions.

### Effect of parameter adjustment on SourceTracker performance

In order to evaluate effect of parameter adjustment on SourceTracker performance, additional three specific artificial configurations (configurations A, B, and C) covering both negative and positive sources were run by different parameter settings. Changes in parameters away from default conditions had variable effect on SourceTracker performance, but mainly depending on the percentage of source present within the sink (Additional file 1: Table S5). Alteration in restart (20, default = 10) and burn-in (1000, default = 100) resulted in similar RSD profile as default condition. Increasing rarefaction depth to 50,000 (default = 1000) consistently decreased RSD in identifying ratio of true positive sources (i.e., WA and NT) in all three configurations, while it did not improve in detecting true negative sources (i.e., HF and AF). Changes in α and β Dirichlet hyperparameters had variable effect. Decrease in RSD was observed in configuration A by all alterations of α and most alterations of β (except β=0.004, 0.006, 0.08, and 0.1), with the lowest RSD achieved by α=0.01 (RSD of WA and NT ≤1%). On the contrary, in configurations B and C, alterations of α and β were more likely to be accompanied by increase in RSD, with lowest RSD (≤ 10%) by α = 0.1/β = 0.04 in configuration B and α=0.001/0.05 in configuration C. Noteworthy, with the exception of β = 0.002/0.004/0.006 in configuration A, α = 0.00001/β = 0.008 in configuration B, and α = 0.05/β = 0.002 in configuration C, both default and other parameter alterations tended to detect true negative sources as positive at extremely low level. The excellent improvement in identifying true negative sources by these specific α and β values significantly increased sensitivity, specificity, precision, and accuracy, which, however, did not necessarily associated with low RSD. Overall, such a variable parameter effect observed here emphasized the need for comprehensive parameter optimization by exquisite experimental design on regional source-sink analysis.

### Indicator ARGs

**Fig. 4:**
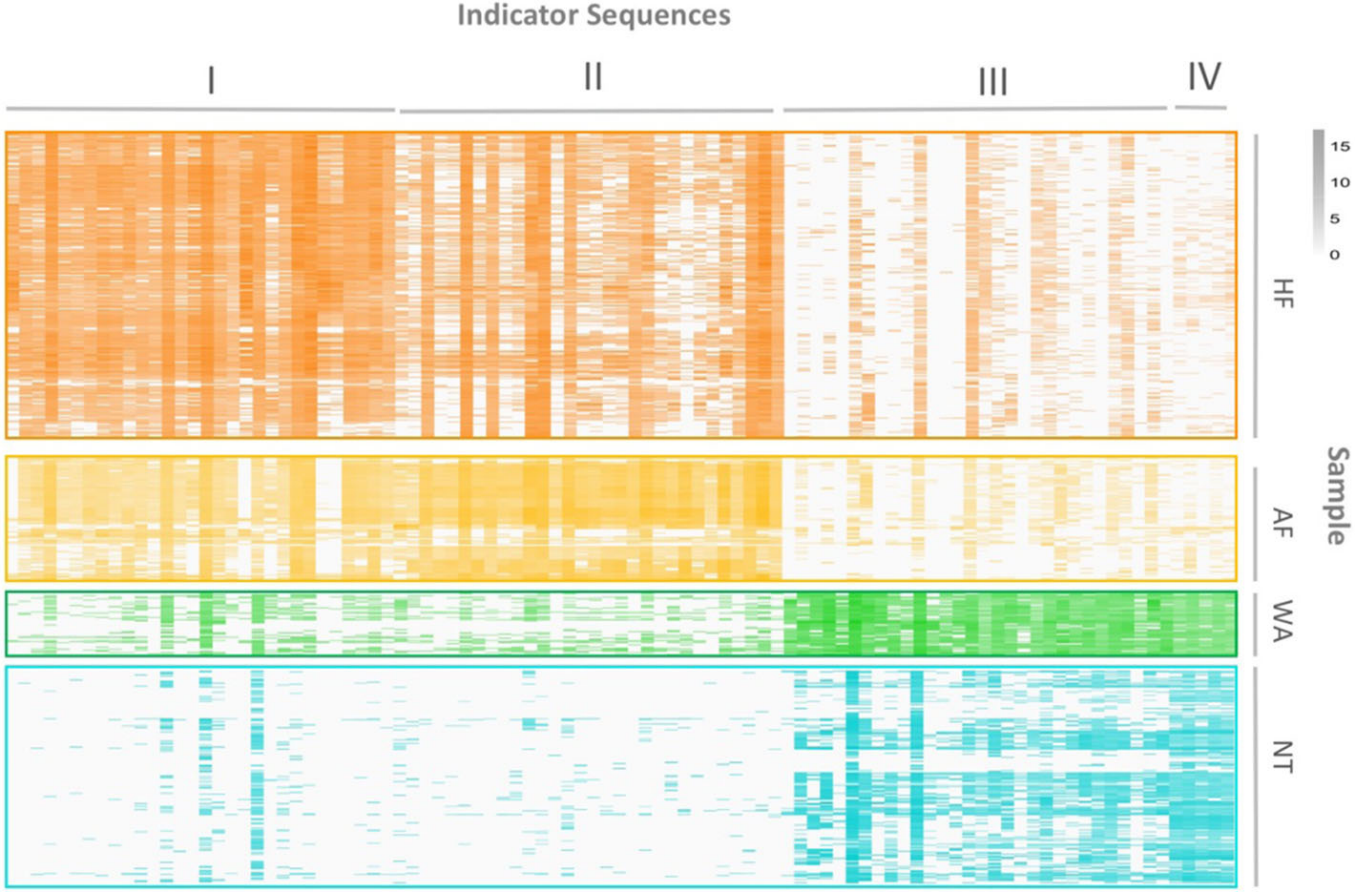
Abundance profile (log2 transformed) of 95 indicator ARGs in all downloaded metagenomic datasets. In the heatmap, four groups of indicator ARGs were arranged from left to right: I. 30 HF indicator ARGs; II. 30 AF indicator ARGs; III.30 WA indicator ARGs; IV. Five NT indicator ARGs. The four heatmap blocks from top to bottom were abundance profile of all indicator ARGs in HF, AF, WA, and NT

Besides characterizing patterns of overall ARG profiles across such broad range of environments, we expanded this study by identifying representative ARGs belonging to each ecotype and thereby lending insight into the potential roles of these ARGs in shaping resistome. Although a few studies have applied representative ARGs to distinguish one environment from another [14, 15, 37, 38], they were conducted at one or a handful of fixed locations and focused on a few typical ARGs such as *sul*1 and *tet*W. In the study here, large-scale profiling of 3502 detected ARGs across 656 diverse samples could help find out more solid indicators using robust statistical method. Based on ARG distribution across the four ecotypes, 95 ARGs were chosen as indicators (Fig. 4, Additional file 1: Table S6), including 30 indicators in each of HF (IV≥0.56, *P* < 0.001 (IV, indicator value)), AF (IV≥0.68, *P* < 0.001), and WA (IV≥0.65, *P* < 0.001). Considering much lower abundance profile in NT samples, only 5 ARGs were selected as indictors of the ecotype (IV≥0.34, *P* < 0.001). Different ecotypes were characterized by different ARG indicators, e.g., (1) HF indicators were mainly composed by resistance genes against beta-lactam (class A), vancomycin, bacitracin, and tetracycline; (2) AF were tetracycline, aminoglycoside, and MLS; (3) WA were bacitracin, MLS, and beta-lactam; and (4) NT was multidrug. Abundance distribution of these indicators clearly implied much higher abundance level in each indicated ecotype while lower in others. Although indicators were a minor part of total 3502 detected ARGs, they corresponded to the dominant among ARGs detected in each ecotype, e.g., comprising up to 11, 20, and 17% of ARG abundance in HF, AF, and WA, respectively. Specifically, top abundant indicators were *bac*A (bacitracin), class A beta-lactamase (beta-lactam), and *van*R (vancomycin) in HF, *aad*E (aminoglycoside), *mef*A (MLS), *tet*40 (tetracycline) in AF, *cpx*R (transcriptional regulatory), *arr* (rifamycin), *omp*R (multidrug) in WA, and *mex*F (multidrug) in NT. These top abundant indicators were dominant groups in each ecotype, indicating their key roles in shaping resistome and driving fluctuation. In addition, in a graph of indicator, relative abundance of ARGs in samples where they occur vs occupancy (Additional file 2: Figure S4), a few indicator ARGs appeared to be generalists, with high relative abundance across a large number of samples outside their indicated ecotypes, e.g., WA indicator *omp*R (multidrug) was detected in all WT samples meanwhile detected in 76% non-WT samples and HF indicator *bcr*A (bacitracin) detected in 99% HF samples meanwhile detected in 73% non-HF samples. Some indicators tended towards specialists, with high relative abundance but detected in fewer samples outside indicator ecotypes, e.g., class A beta-lactamase (beta-lactam) in HF and *ere*A (MLS) in WA were detected in less than 5% other samples. In addition to specific indicators, five ARGs with relatively high *R*^2^ correlation (≥ 0.50) with total ARG abundance across all samples were chosen as the general indicators (Additional file 2: Figure S5, Additional file 3: Table S7), including three ARGs of *mex*X and *acr*B with multidrug resistance, one ARG in class C beta-lactamase, and one unclassified ARG coding alamin adenosyltransferase.

### Common and unique ARGs

**Fig. 5:**
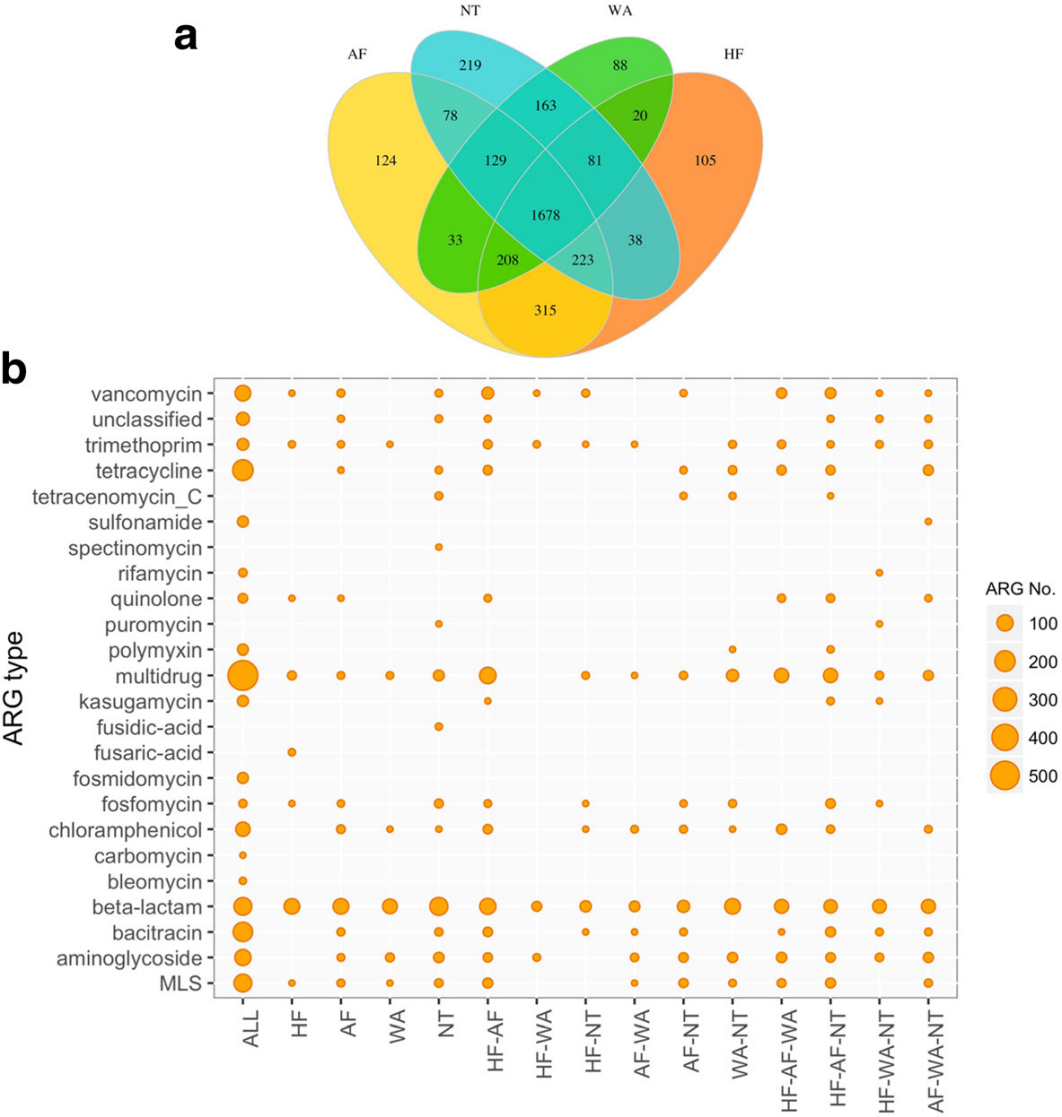
Common and unique ARGs across four ecotypes. **a** Venn diagram. **b** Sequence number distribution of common/unique ARG groups (*X*-axis, e.g., HF–AF indicated common ARG group shared by HF and AF ecotypes) across 24 ARG types (*Y*-axis)

According to occurrence among samples, all 3502 detected ARGs were classified as the unique or common in each or combination of the four ecotypes (Fig. 5). 1678 ARGs were shared among all ecotypes (i.e., detected in at least one sample in each ecotype) mainly belonging to resistance genes against multidrug (*n* = 562), tetracycline (*n* = 202), bacitracin (*n* = 179), beta-lactam (*n* = 137), MLS (*n* = 137), and aminoglycoside (*n* = 101). These common ARGs made up a large percentage of ARGs detected in each ecotype, e.g., 60–68% in HF, AF, and AS; and 74% in NT. Such large amount of widespread ARGs indicated frequent flow crossing ecological barriers, which has been detected between habitats, such as soil and human [39], WWTP and surface water [40, 41], as well as livestock farm and surrounding environments [42]. Interestingly, 86 of 95 specific indicator ARGs were shared by all ecotypes, whereas the other 9 indicators were all three-ecotype common ARGs. Among ARGs common between specific two ecotypes, HF and AF (hereafter refer to HF-AF (common between ecotypes indicated by ‘ecotype-ecotype’)) shared most ARGs (*n* = 315) followed by NT-WW (*n* = 163) and FA-NT (*n* = 78), least shared by FH-WW (*n* = 20), FA-WW (*n* = 33), and FH-NT (*n* = 38). Especially, the top three in HF-AF were resistance genes against multidrug, beta-lactam, and vancomycin. In addition, the two feces ecotypes, HF and AF, shared 223 ARGs with NT and 208 ARGs with AS. Regarding unique ARGs in each ecotype, most unique ARGs were detected in natural (*n* = 219) while least in WWTP (*n* = 88). Indeed, functional metagenomics have resulted in elucidating entirely new resistance functions in natural environments [26, 39, 43], implying substantial potential of underappreciated wild resistome in contributing to future health risks. On the contrary, WWTPs were engineered facilities of wastewater mixture, featuring in active exchange of existing genes from various human-related sources [5, 44].

## Discussion

Because of multi-sources interaction and regional biogeographical characters, directly identifying sources of contamination and implementing targeted mitigation strategies have long been a challenging topic in AMR control. Based upon explicit evaluation of potential and limitation, in the current big-data era, we here presented a novel framework combining both metagenomic profiling and machine-learning classification SourceTracker to address source tracking of ARG contamination in the environment. Through comprehensive performance examination by both global-scale and region-specific datasets, feasibility of the platform was generally well supported despite of its fluctuation in predicting low source ratio. However, it should be noted that predicted source proportions from SourceTracker were limited by the comprehensiveness of source datasets used for training, thus, sample impurity and regional variation among datasets retrieved from public databases might bias our analysis to an extent despite effort in smoothing out dataset heterogeneity. For example, more HF and NT samples along with less AF and WA samples, might hinder SourceTracker in identifying discriminatory ARG signatures in AF and WA to attribute sources. We believe that large-scale metagenomic projects of either pristine or human-impacted environments will dramatically expand availability of more representative resources. In particular, training by region-specific datasets will improve its source discrimination performance in a target area.

Indeed, ARG pollution is a region-specific problem with global impact. Both anthropogenic activities and geographic features can influence ARG pollution status substantially. ARGs can enter environments through a variety of pathways, including point sources such as discharge from WWTPs and livestock farms, as well as non-point sources such as runoff from fields treated with biosolids or manure. In contrast to direct release of pollutants into target environment by point sources, contamination caused by non-point sources is always subject to dilution and decay thus largely impeding accurate source prediction. Additionally, cross-reactivity between source pollution and environmental background will generate certain biotic and abiotic conditions favoring specific bacteria and/or genes [11, 12, 45, 46]. What might further confuse source detection is frequent genetic exchange, that is, once associated with efficient mobile genetic elements like broad-host-range plasmids [47, 48] and class I integrons [49, 50], dissemination of ARGs across phylogenetic and ecological barriers could be dramatically enhanced. Moreover, resistome development was even more complicated by diverse co-selection pressure (e.g., metal and biocides) in source/sink environment [51, 52]. Under such complex environmental conditions, source-tracking investigations can only be achieved through comprehensive biogeographical surveillance. Experiments directed to identifying quantitative source-sink relationship at certain sites should be carefully designed to normalize possible background influence, such as comprehensive analysis along temporal (e.g., dry and wet seasons) and spatial (e.g., upstream and downstream) scales. Also, high sensitivity and specificity of prediction platform are required for disentangling such complicated source-sink relationship. Through integrating metagenomics profiling with machine learning classification, excellent source prediction has been demonstrated in this study, which is far beyond the capability of traditional source-tracking methods. In particular, metagenomic profiling improves source tracking through parallel detection of a multitude of different genetic markers that are unique to sources, and machine learning classification algorithm deemphasizes overlapped signatures that occur among training sets to further minimize biases like background cross-reactivity. Compared with traditional methods, broad-spectrum ARG profiling-based SourceTracker classification took a fundamental step in advancing precise source-sink relationship quantification. To the best of our knowledge, this is the first study directed to combine broad-spectrum ARG profiles and machine-learning classification to track potential ARG pollution sources.

To further explore potentials and limitations of the approach in source tracking, especially its application in diluted areas such as recreational beaches in which correct source identification is crucial in public health risk assessment, more extensive parameter optimization and rigorous in-laboratory test are necessary. As demonstrated in source discrimination of artificial configurations, SourceTracker will report high variability in the proportion estimates for low-representative source. Therefore, confident results of low source proportion should be based on multiple runs instead of single run, which is consistent with the previous studies on community structure-based fecal signal tracking [22, 33]. Alteration of the investigated parameters resulted in RSD variation in contrast to default setting. Increasing rarefaction depth consistently decreased RSD, but no equivalent improvement in sensitivity and specificity was observed, which suggested that increasing rarefaction depth only enhanced repeatability due to the inclusion of 50-fold more ARG abundance for SourceTracker analysis. By assigning different relative values for α and β, prior counts (relative to the number of sequences in the test sample) that smooth the distributions for low-coverage source and sink samples were adjusted, which had a remarkable effect on detecting true negative sources. However, in which (in)dependent way the two parameters of prior count affecting SourceTracker performance still need more rigorous examination. When applying the classification tool in source tracking of complex region-specific pollution, both complementary in vitro assay and thorough assessment of parameter settings should be conducted to enhance its performance in identifying true/false positive sources and detecting known proportions of sources present within a sink matrix. In this way, its capability in detecting low level of ARGs will be fully realized, lending much more confidence in broad application.

In addition to exploring overall ARG profile in discriminating source-sink relationship, we extended this study to identify representative ARGs in characterizing distinct ecotypes. Realizing challenges in predicting attenuated source-sink signal, indicator analysis was performed to seek for additional tools. The generalist and specialist indicator ARGs act as representative ARG signatures in the indicated ecotype, providing potential in resolving closely connected ecosystems through coupling with general abundance profile-based source tracking. One typical example is coastal site in which continuous gradient pollution is hard to be detected by overall abundance distribution. As mixing occurred across the coastal margin, a high level of abundance across many environments might be maintained for generalist indicators, while pattern specific to their respective indicated environment were more likely associated with specialist indicators [53]. In addition, among retrieved common and unique ARGs in such global-scale samples, large part of overlapped ARGs implied past frequent transmission, whereas minor unique ARGs might be intrinsic to each ecotype. We believe that region-specific studies will help identify more representative ARG signatures and thereby contribute to assessing resistome development in the area.

## Conclusions

Altogether, by combining comprehensive metagenomic ARG profiling with machine-learning classification SourceTracker, we here presented a novel quantitative framework to address ARG pollution source tracking. Although sequencing expense and computational complexity might impede the platform application as a routine ARG pollution monitor tool, continued reduction in sequencing costs and increase in public accessible computational resources (e.g., online ARG annotation platform ARGs-OAP [54]) may soon make this approach feasible. Following predicted source contribution status, risk ranking of different sources in ARG dissemination will be possible, thereby paving the way for establishing priority in mitigating further ARG spread. Particularly, differentiation of sources will shed light on areas where intervention can be most effective in reducing ARG spread in the environment. Thus, the presented source-tracking platform will have far-reaching significance for both science community and public authorities in AMR control.

## Methods

### Dataset information

A total of 656 metagenomic datasets covering four distinct ecological categories (i.e., HF, AF, WA, and NT) were included in this study, which were downloaded from public databases including NCBI-SRA (https://www.ncbi.nlm.nih.gov/sra/), MGRAST (http://metagenomics.anl.gov/), and HMPDACC (http://hmpdacc.org/HMIWGS/all/) during period from November 2016 to January 2017. In order to guarantee data quality, all datasets were generated on Illumina shotgun sequencing platform and downloaded in FASTQ format with original sequencing quality information. In addition, background information of all datasets was supported by relevant publications. To minimize possible bias introduced by dataset heterogeneity, only feces samples of healthy human adults were used. Considering large variation in microbial communities at different wastewater treatment processes, activated sludge was used as the representative of WWTP samples. All downloaded raw data went through quality check and filtration by PRINSEQ (prinseq-lite.pl using parameter setting: mean quality score ≥ 20 and number of ambiguous ≤ 1). To eliminate inconsistence in sequences, only reads with length ≥ 100 bp were included and then all trimmed to 100 bp. In final datasets, average sequence number across all samples was 27,712,728 with minimum 2,038,492 and maximum 210,543,839. The full list of sample information was summarized in Additional file 1: Table S1.

### ARG annotation and community structure retrieval

Potential ARGs in all datasets were retrieved through pipeline embedded in online platform ARGs-OAP (smile.hku.hk/SARGs) [54]. Briefly, pre-screening for ARG-like and 16S rRNA gene sequences were conducted by UBLAST using Perl script supplied by the platform. The candidate ARG sequences were aligned against ARG database SARG using BLASTX and then classified according to the SARG hierarchy (type-subtype-sequence) when meeting the criteria in BLASTX results (i.e., alignment length 25 aa, similarity 80% and evalue 1e-5). ARG abundance (unit: copies of ARG per copy of 16S rRNA) in each metagenomic dataset was ARG-like sequence number normalized to the corresponding ARG reference sequence length (nucleotide) and the number of 16S rRNA genes. Community composition was identified by 16S rRNA gene hypervariable region from metagenomic datasets by USEARCH against Greengenes nr90 database.

### SourceTracker method validation

Analysis was conducted in R using SourceTracker under default parameter settings (burnin = 100, nrestarts = 10, ndraws.per.restart = 1, delay = 10, α = 0.001, β = 0.01, rarefaction_depth = 1000), in which different categorical probabilities were used for calling a certain ratio of source present. The predictive performance of the classifier in ARG source tracking was evaluated by leave-one-out cross-validation of 656 datasets with five runs. For each sample, predicted proportion for each of five potential sources (i.e., HF, AF, WA, NT, and UN (unknown)) across all five runs was averaged and source with the highest average proportion was deemed as the predicted source. Consistency between the predicted source and original ecotype was used to calculate general SourceTracker prediction accuracy. Within each ecotype, standard deviation (SD) and RSD were calculated across predicted proportions. To further examine potential and limitation of SourceTracker in predicting specific source contributions within sink samples, eight artificial sink configurations were generated containing defined proportions of source ARGs. ARG tables consisted of average proportions of ARGs associated with each ecotype were combined into a single representative source sample. The sink sample was generated by multiplying and adding these averages into a single configuration. The SourceTracker output was designated as the ‘predicted’ proportion, and the artificial source inputs were designated as ‘expected’. Taking variation between runs (i.e., RSD) into account, predicted proportions were compared with the expected across configurations. In addition, the trained classifier was challenged by three sets of metagenomic samples with obvious gradient influence from human activities to evaluate its performance in the following real application: 1). influent and effluent samples of a WWTP from summer and winter seasons respectively [55]; 2). marine sediments collected from different HK coastal locations [56]; 3). PRE sediment in south China and deep ocean sediment in SCS [36].

### Evaluation of parameter adjustment

Three particular artificial configurations (configuration A, B, and C), covering a range of positive and negative sources, were applied to evaluate effect of parameter adjustment on SourceTracker performance. HF and AF were included as negative control sources which should not be detected, while WA and NT were present at defined concentrations which should always be detected. Independent parameter adjustment of rarefaction depth (1000 (default), 5000, 20,000 and 50,000), burn-in period (100 (default) and 1000), restarts (10 (default) and 20), as well as Dirichlet hyperparameters α (0.1, 0.05, 0.01, 0.005, 0.001(default), 0.0005, 0.0001, 0.00005, 0.00001) and β (0.1, 0.08, 0.06, 0.04, 0.02, 0.01(default), 0.008, 0.006, 0.004, 0.002) was investigated. Based on predicted presence/absence and ratio of sources in the configurations, sensitivity (TP/(TP + FN)), specificity (TN/(TN + FP)), precision (TP/(TP + FP)), accuracy ((TP + TN)/total number of sources) and RSD were calculated to evaluate effect of the parameter adjustment on SourceTracker prediction performance (TP: true positive, detected WA and NT sources; TN: true negative, non-detected HF and AF sources; FP: false positive, detected AF and HF sources; FN: false negative, non-detected WA and NT sources).

### Statistical analysis

The beta-diversity of ARG and community structure between different samples was compared using principal coordinate analysis (PCoA) based on Bray-Curtis distance. Procrustes test for correlation analysis between ARGs and bacterial communities was performed in R with the vegan package. To identify specific indicator ARGs that characterize each of the environments, both abundant (this is called specificity) and predominant (this is called fidelity) in the type of environment, the package labdsv and test indval were run in R, where IV ranges from 0 to 1 with higher values for stronger indicators. Linear correlation was conducted to identify the association between the abundance distribution of individual profile of each ARG and general profile across all ARGs, in which those ARGs with higher linear correlation were selected as potential general indicators of overall ARG pollution. Common and unique ARGs were obtained based on absence and presence pattern in the four ecotypes. All graphs were produced by ggplot2 package. R script used for this study is available at https://github.com/LiguanLi/SourceTrack.

## Additional files

Additional file 1: **Table S1.** Metadata of 656 metagenomic datasets. **Table S5.** SourceTracker parameter adjustment. **a** Defined source input ratio in the three artificial configurations (A, B, and C). **b** Effect of parameter adjustment on SourceTracker prediction performance of configurations A, B, and C (indicated by RSD, sensitivity, specificity, precision, and accuracy). Table S6. Statistical details (IV and *P* value) of 95 indicator ARGs of the four ecotypes. (XLSX 78 kb)

Additional file 2: **Figure S1.** ARG abundance profile-based PCoA across all collected metagenomics datasets (featured by both their ecotype and project/study). Shape of each dot indicates different ecotype, and dot color indicates different project or study in which these datasets involved. **Figure S2.** PCoA analysis based on abundance profiles of overall ARG (**a**) and community structure at phylum level (**b**). Procrustes analysis revealed that PCoA of overall ARG and community structure profiles are significantly correlated (*P* < 0.001, based on 9999 permutations). **Figure S3.** Predicted source proportion in WWTP influent and effluent by SourceTracker. **Figure S4.** Occurrence and abundance profile of indicator ARGs. **a** Relative abundance of indicator ARGs in samples where they occur vs occupancy. **b** Specific occurrence (occurrence ratio in samples of indicated ecotype) vs general occurrence (occurrence ratio in samples outside indicated ecotype) of indicator ARGs across 656 samples. **Figure S5.** Abundance profiles (log2 transformed) of five top ARGs with high correlation with overall abundance across 656 metagenomic datasets. Inner circles, top correlation sequence I–V (in an outward direction from innermost circle layers); outer circle, overall ARGs abundance. (DOCX 2108 kb)

Additional file 3: **Table S2.** SourceTracker prediction proportion variation (indicated by mean, SD, and RSD) between runs in leave-one-out cross-validation by 656 samples (refer to Fig. 1d). **Table S3.** SourceTracker prediction proportion variation (indicated by mean, SD, and RSD) between runs of eight artificial configurations (refer to Fig. 2). **Table S4.** Location of 12 HK sediment samples, 9 PRE and SCS sediments. **Table S7.** Sequence information of ARGs of relative high correlation (*R*^2^ ≥ 0.5) with overall abundance profiles. (DOCX 1430 kb)

## Abbreviations

AF: Animal feces
AMR: Antimicrobial resistance
ARG: Antibiotic resistance gene
HF: Human feces
HK: Hong Kong
HTS: High-throughput sequencing
IV: Indicator value
MLS: Macrolides-lincosamides-streptogramines
NT: Natural environments
PRE: Pearl River Estuary
RSD: Relative standard deviation
SCS: South China Sea
SD: Standard deviation
UN: Unknown
WA: Activated sludge from wastewater treatment plant
WWTP: Wastewater treatment plant
