# Supplementary material for: Tracking antibiotic resistance gene pollution from different sources using machine-learning classification
Source: Microbiome. 2018 May 24;6:93. doi: 10.1186/s40168-018-0480-x (PMC5966912; doi:10.1186/s40168-018-0480-x)
Supplement: Supplementary file 2 — Figure S1. ARG abundance profile-based PCoA across all collected metagenomics datasets (featured by both their ecotype and project/study). Shape of each dot indicates different ecotype, and dot color indicates different project or study in which these datasets involved. Figure S2. PCoA analysis based on abundance profiles of overall ARG (a) and community structure at phylum level (b). Procrustes analysis revealed that PCoA of overall ARG and community structure profiles are significantly correlated (P < 0.001, based on 9999 permutations). Figure S3. Predicted source proportion in WWTP influent and effluent by SourceTracker. Figure S4. Occurrence and abundance profile of indicator ARGs. a Relative abundance of indicator ARGs in samples where they occur vs occupancy. b Specific occurrence (occurrence ratio in samples of indicated ecotype) vs general occurrence (occurrence ratio in samples outside indicated ecotype) of indicator ARGs across 656 samples. Figure S5. Abundance profiles (log2 transformed) of five top ARGs with high correlation with overall abundance across 656 metagenomic datasets. Inner circles, top correlation sequence I–V (in an outward direction from innermost circle layers); outer circle, overall ARGs abundance. (DOCX 2108 kb) [file 40168_2018_480_MOESM2_ESM.docx]

**Additional File 1**

**Figures**

**Figure S1**. ARG abundance profile based PCoA across all collected metagenomics datasets (featured by both their ecotype and project/study). Shape of each dot indicates different ecotype and dot color indicates different project or study in which these datasets involved.

**Figure S2.** PCoA analysis based on abundance profiles of overall ARG **(a)** and community structure at phylum level **(b)**. Procrustes analysis revealed that PCoA of overall ARG and community structure profiles are significantly correlated (P < 0.001, based on 9999 permutations).

**Figure S3.** Predicted source proportion in WWTP influent and effluent by SourceTracker.

**Figure S4.** Occurrence and abundance profile of indicator ARGs. **(a)** relative abundance of indicator ARGs in samples where they occur vs occupancy; **(b)** specific occurrence (occurrence ratio in samples of indicated ecotype) vs general occurrence (occurrence ratio in samples outside indicated ecotype) of indicator ARGs across 656 samples.

**Figure S5.** Abundance profiles (log2 transformed) of five top ARGs with high correlation with overall abundance across 656 metagenomic datasets. Inner circles: top correlation sequence I-V (in an outward direction from innermost circle layers); outer circle: overall ARGs abundance.


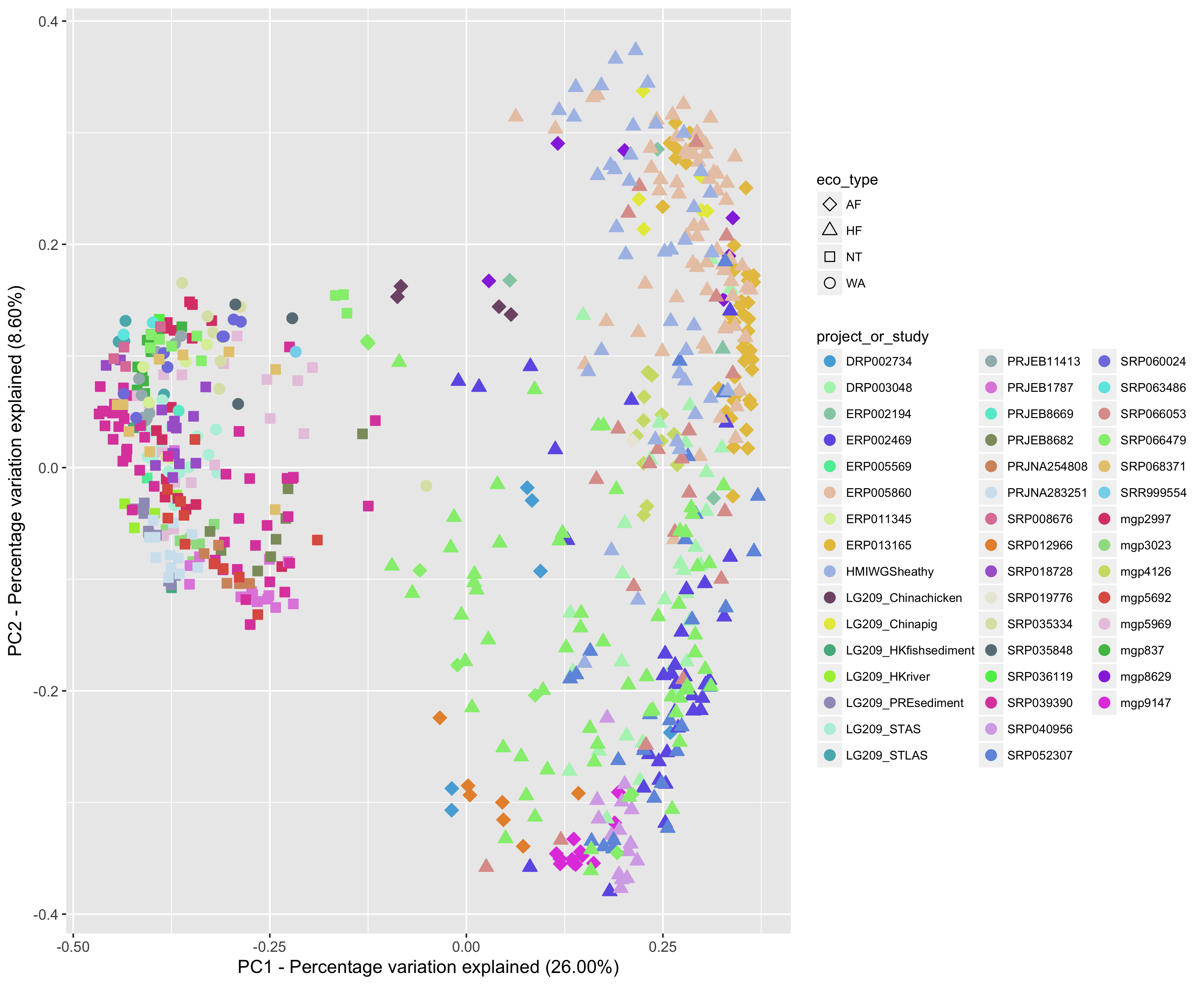


**Figure S1**. ARG abundance profile based PCoA across all collected metagenomics datasets (featured by both their ecotype and project/study). Shape of each dot indicate different ecotype and dot color indicates different project or study in which these datasets involved.


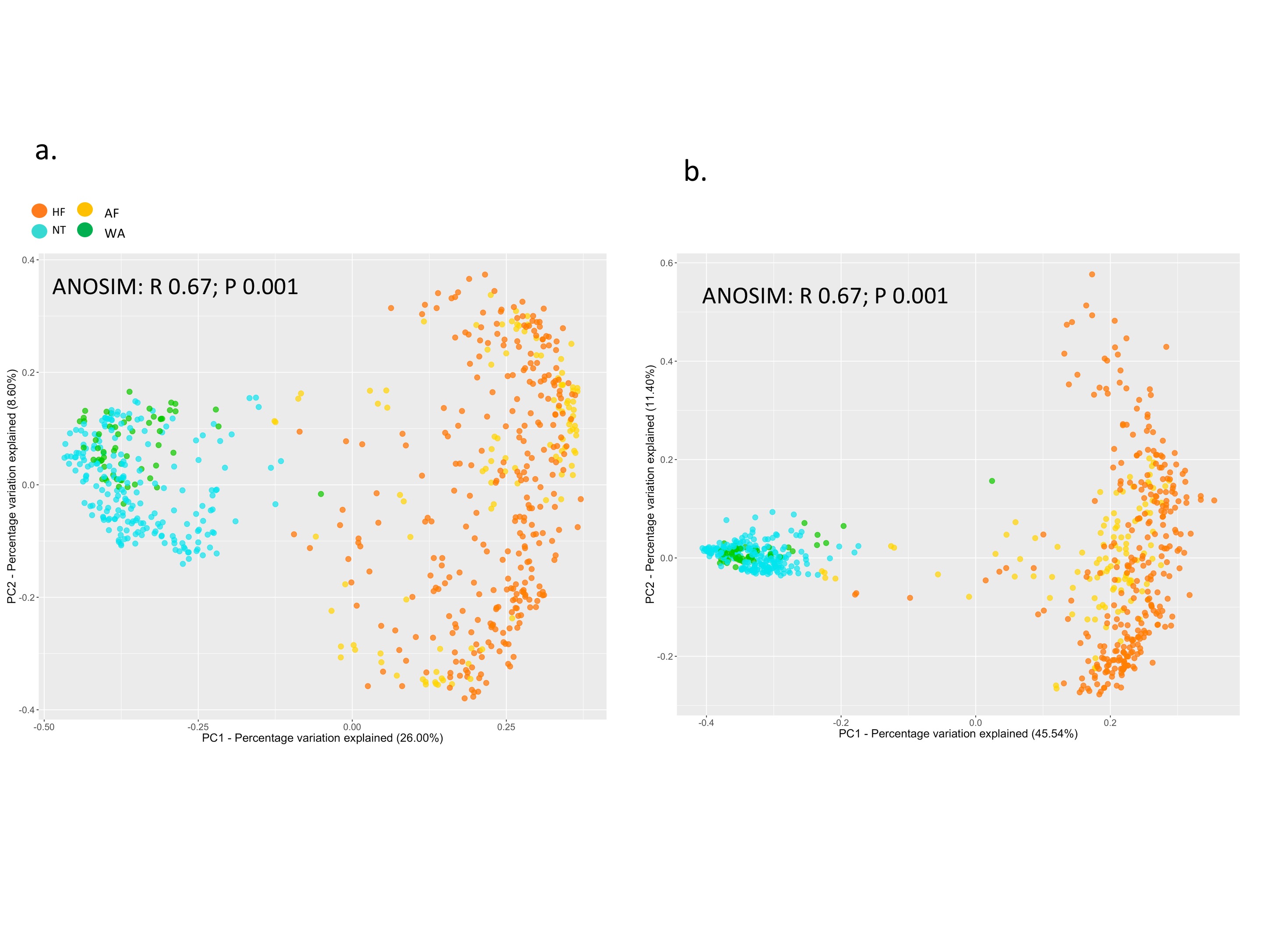


**Figure S2.** PCoA analysis based on abundance profiles of overall ARG **(a)** and community structure at phylum level **(b)**. Procrustes analysis revealed that PCoA of overall ARG and community structure profiles are significantly correlated (P < 0.001, based on 9999 permutations).


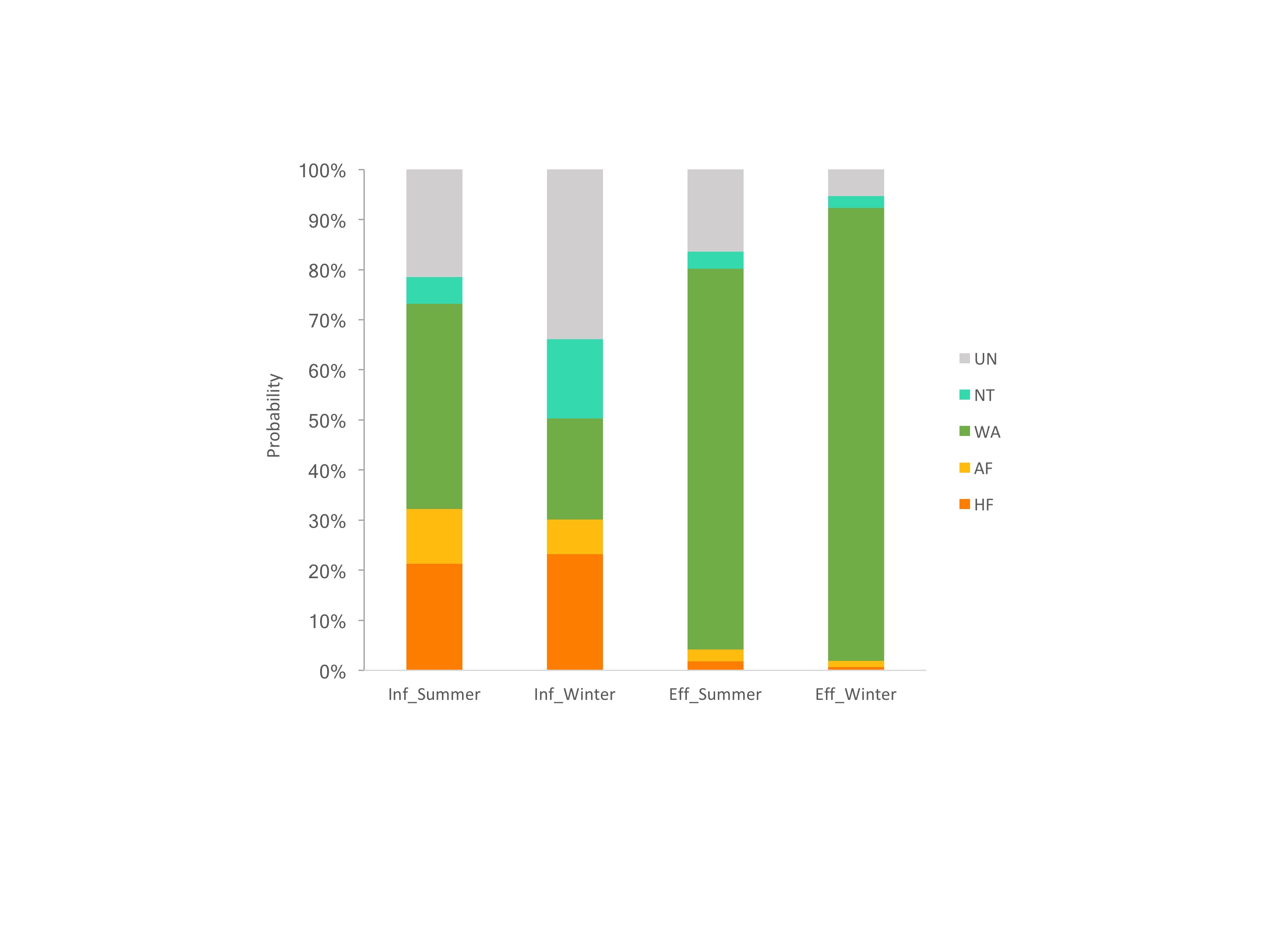


**Figure S3.** Predicted source proportion in WWTP influent and effluent by SourceTracker.


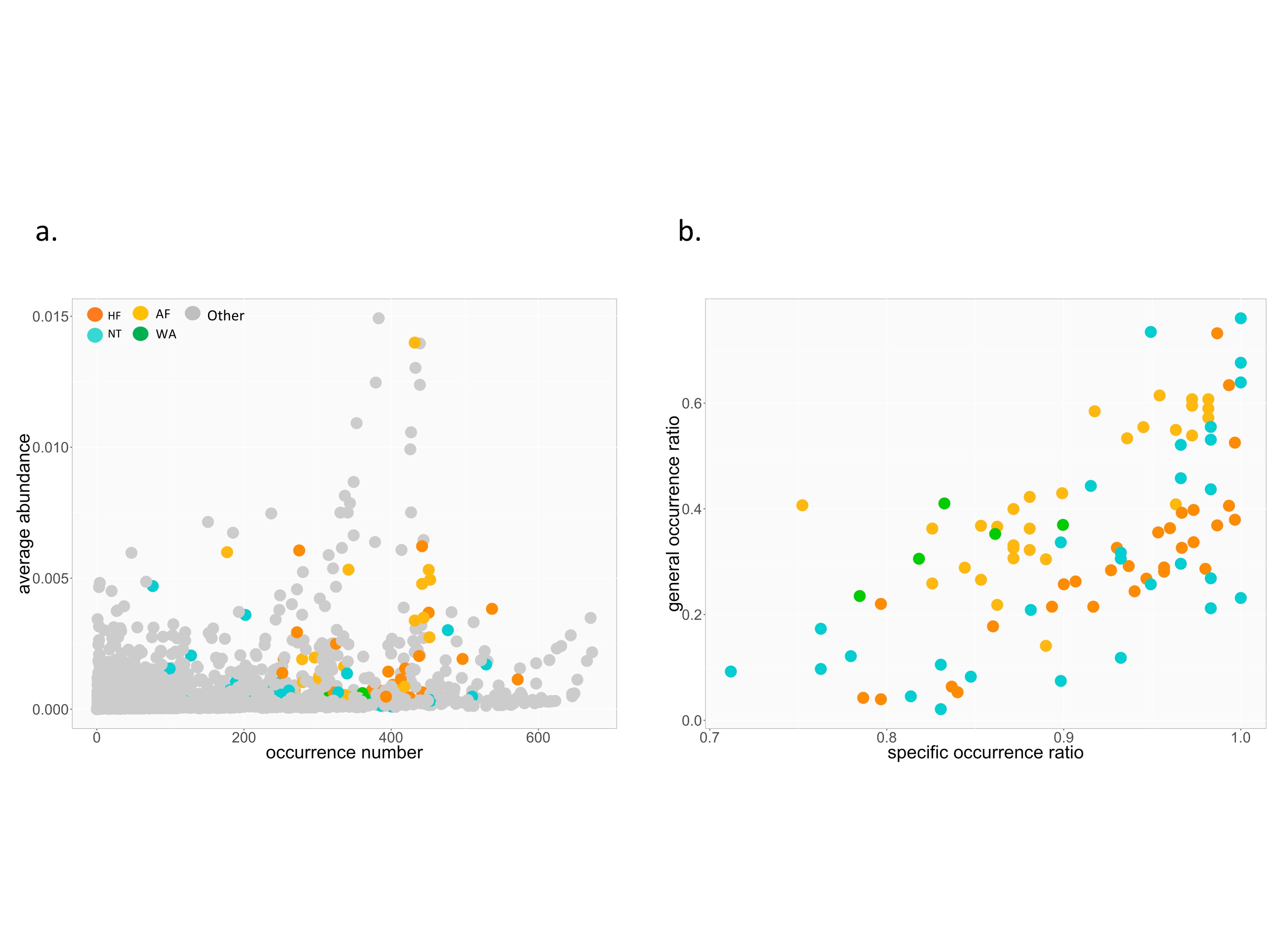


**Figure S4.** Occurrence and abundance profile of indicator ARGs. **(a)** relative abundance of indicator ARGs in samples where they occur vs occupancy; **(b)** specific occurrence (occurrence ratio in samples of indicated ecotype) vs general occurrence (occurrence ratio in samples outside indicated ecotype) of indicator ARGs across 656 samples.


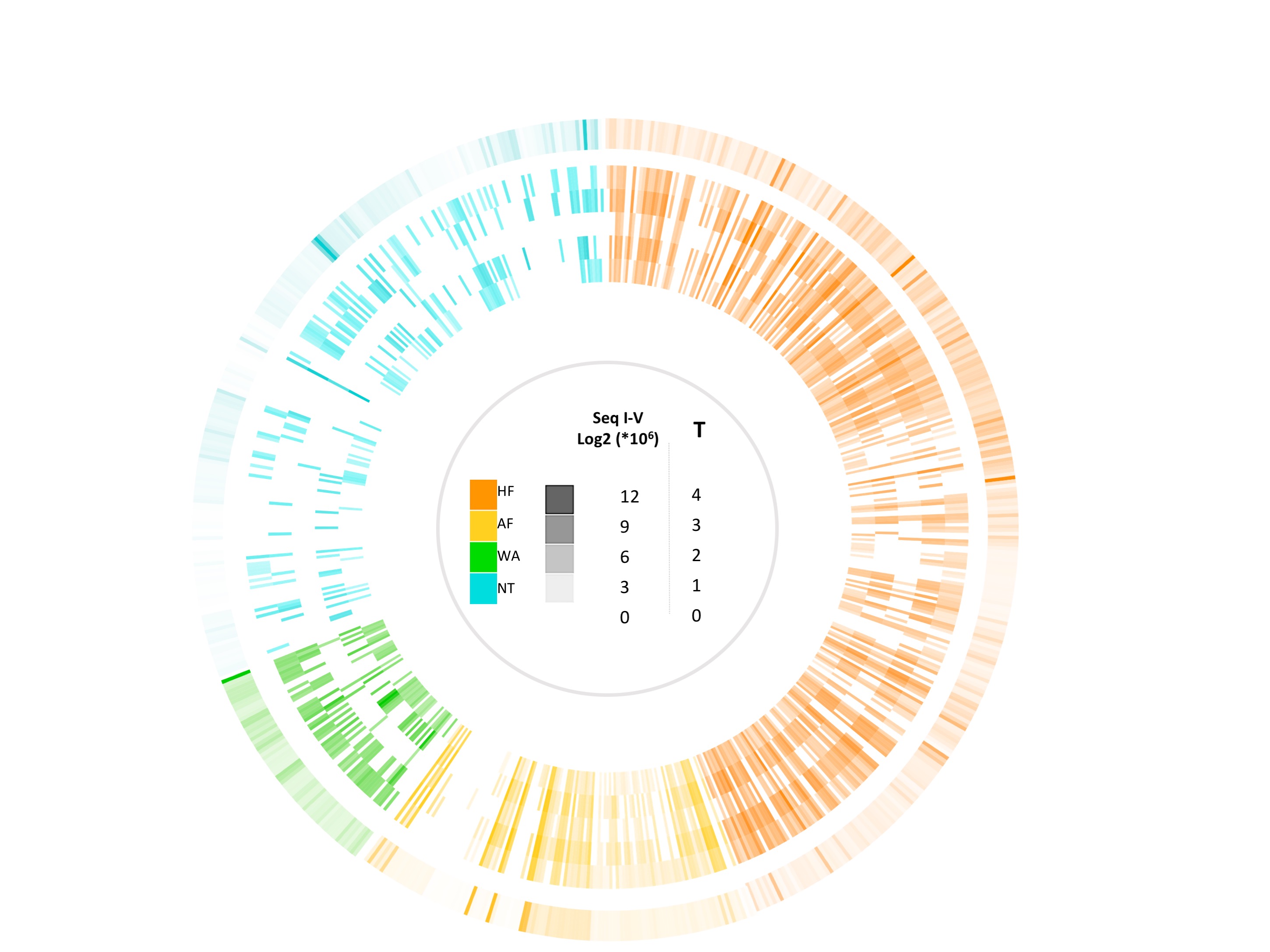


**Figure S5.** Abundance profiles (log2 transformed) of five top ARGs with high correlation with overall abundance across 656 metagenomic datasets. Inner circles: top correlation sequence I-V (in an outward direction from innermost circle layers); outer circle: overall ARGs abundance.
