# Supplementary material for: Tracking antibiotic resistance gene pollution from different sources using machine-learning classification
Source: Microbiome. 2018 May 24;6:93. doi: 10.1186/s40168-018-0480-x (PMC5966912; doi:10.1186/s40168-018-0480-x)
Supplement: Supplementary file 3 — Table S2. SourceTracker prediction proportion variation (indicated by mean, SD, and RSD) between runs in leave-one-out cross-validation by 656 samples (refer to Fig. 1d). Table S3. SourceTracker prediction proportion variation (indicated by mean, SD, and RSD) between runs of eight artificial configurations (refer to Fig. 2). Table S4. Location of 12 HK sediment samples, 9 PRE and SCS sediments. Table S7. Sequence information of ARGs of relative high correlation (R2 ≥ 0.5) with overall abundance profiles. (DOCX 1430 kb) [file 40168_2018_480_MOESM3_ESM.docx]

**Additional File 2.**

**Tables**

**Table S2.** SourceTracker prediction proportion variation (indicated by mean, SD and RSD) between runs in leave-one-out cross-validation by 656 samples (refer to Fig. 1d).

**Table S3.** SourceTracker prediction proportion variation (indicated by mean, SD and RSD) between runs of eight artificial configurations (refer to Fig. 2).

**Table S4.** Location of 12 HK sediment samples, 9 PRE and SCS sediments.

**Table S7.** Sequence information of ARGs of relative high correlation (R^2^ ≥ 0.5) with overall abundance profiles.

**Table S2.** SourceTracker prediction proportion variation (indicated by mean, SD and RSD) between runs in leave-one-out cross validation by 656 samples (refer to Fig. 1D).


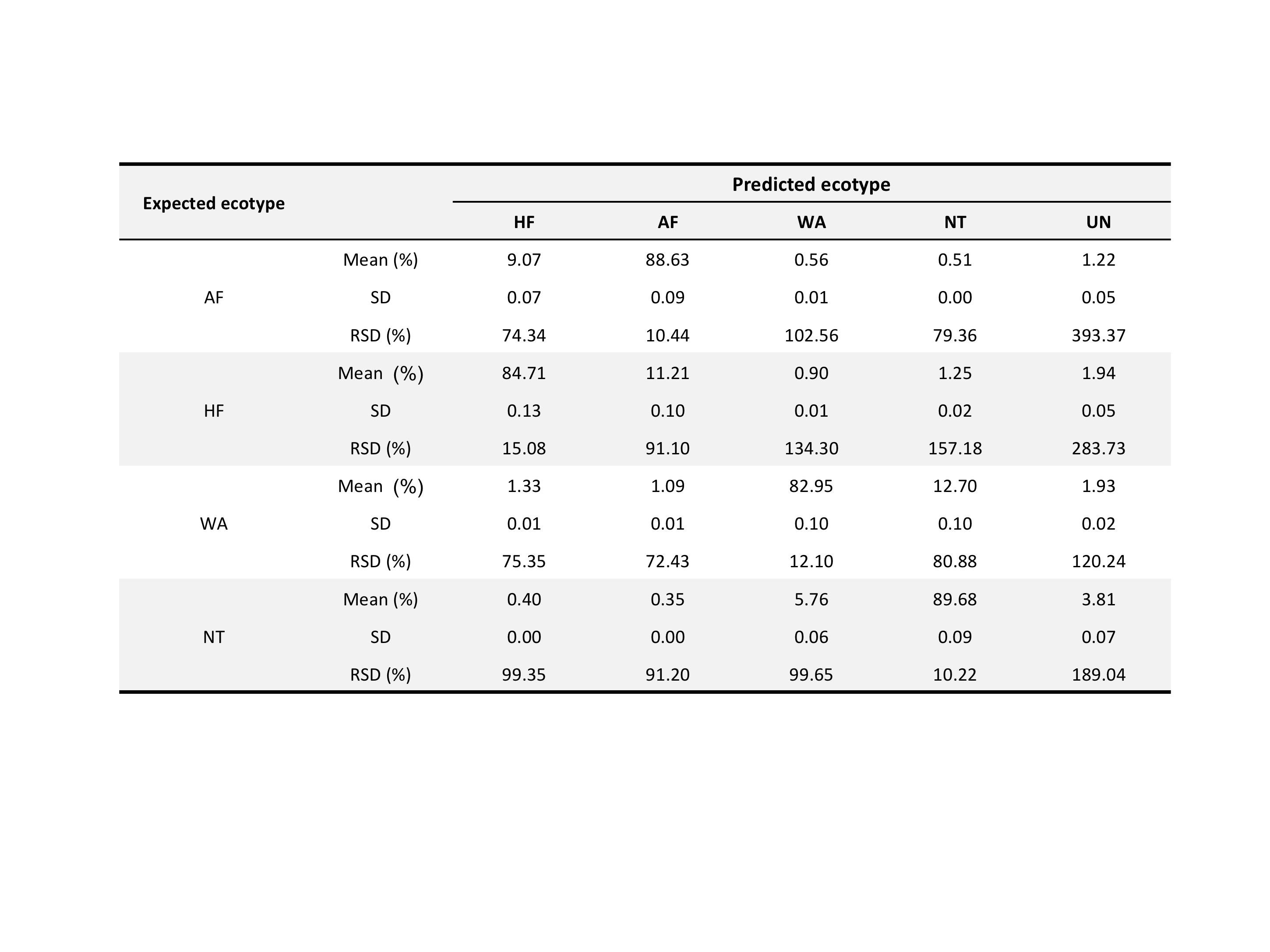


**Table S3**. SourceTracker prediction proportion variation (indicated by mean, SD and RSD) between runs of eight artificial configurations (refer to Fig. 2).


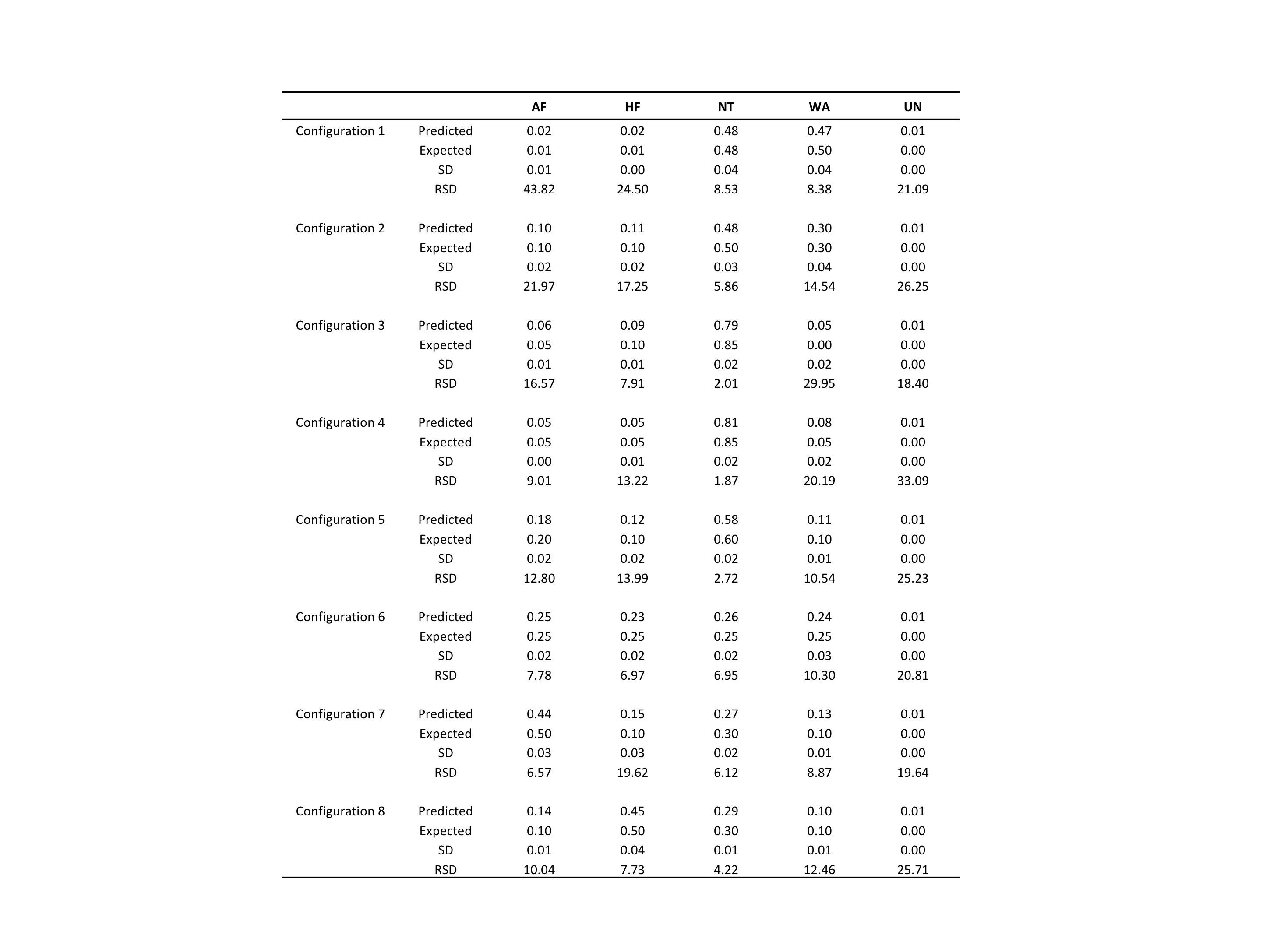


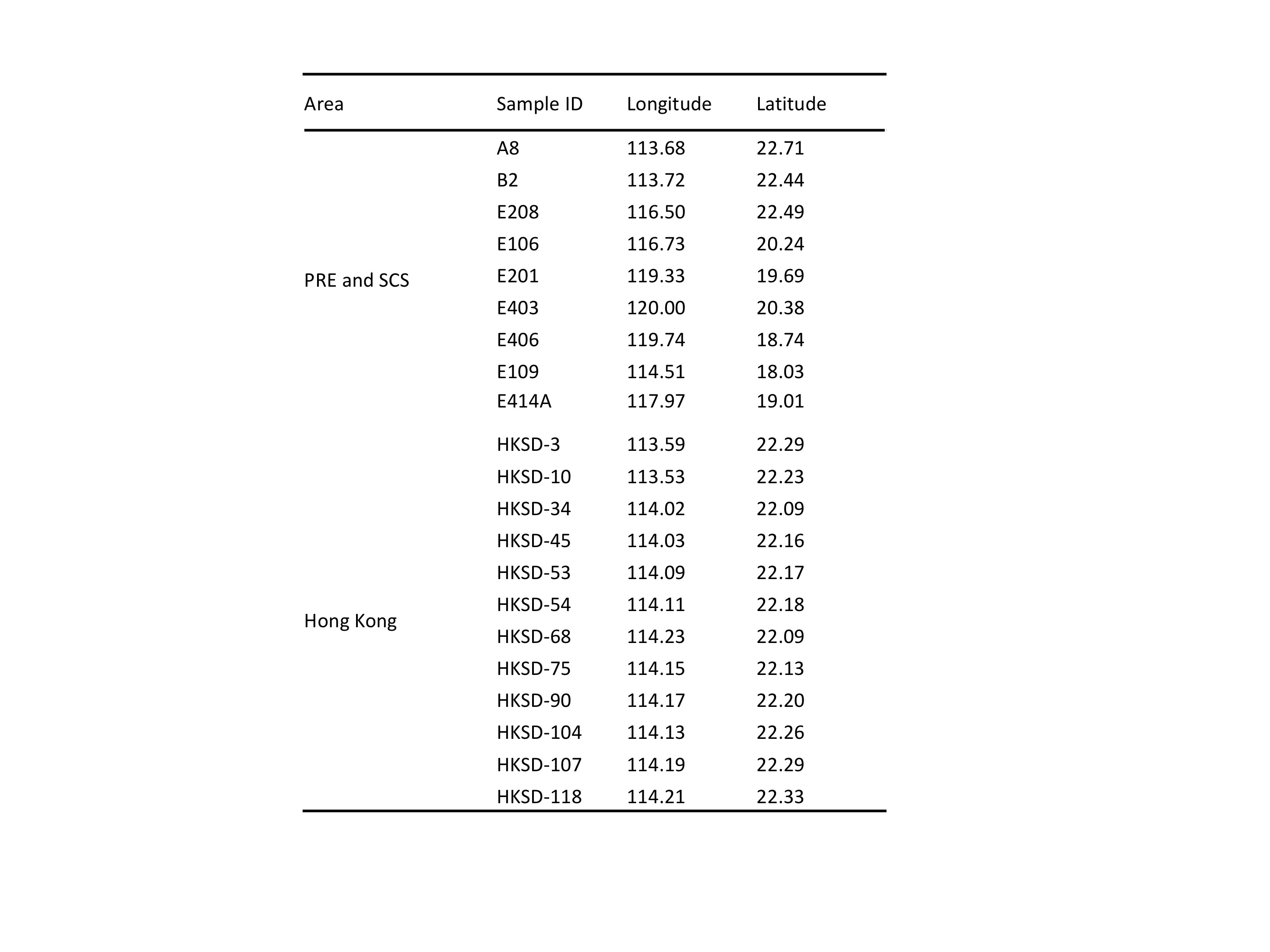
 **Table S4.** Location of 12 HK sediment samples, 9 PRE and SCS sediments.

**
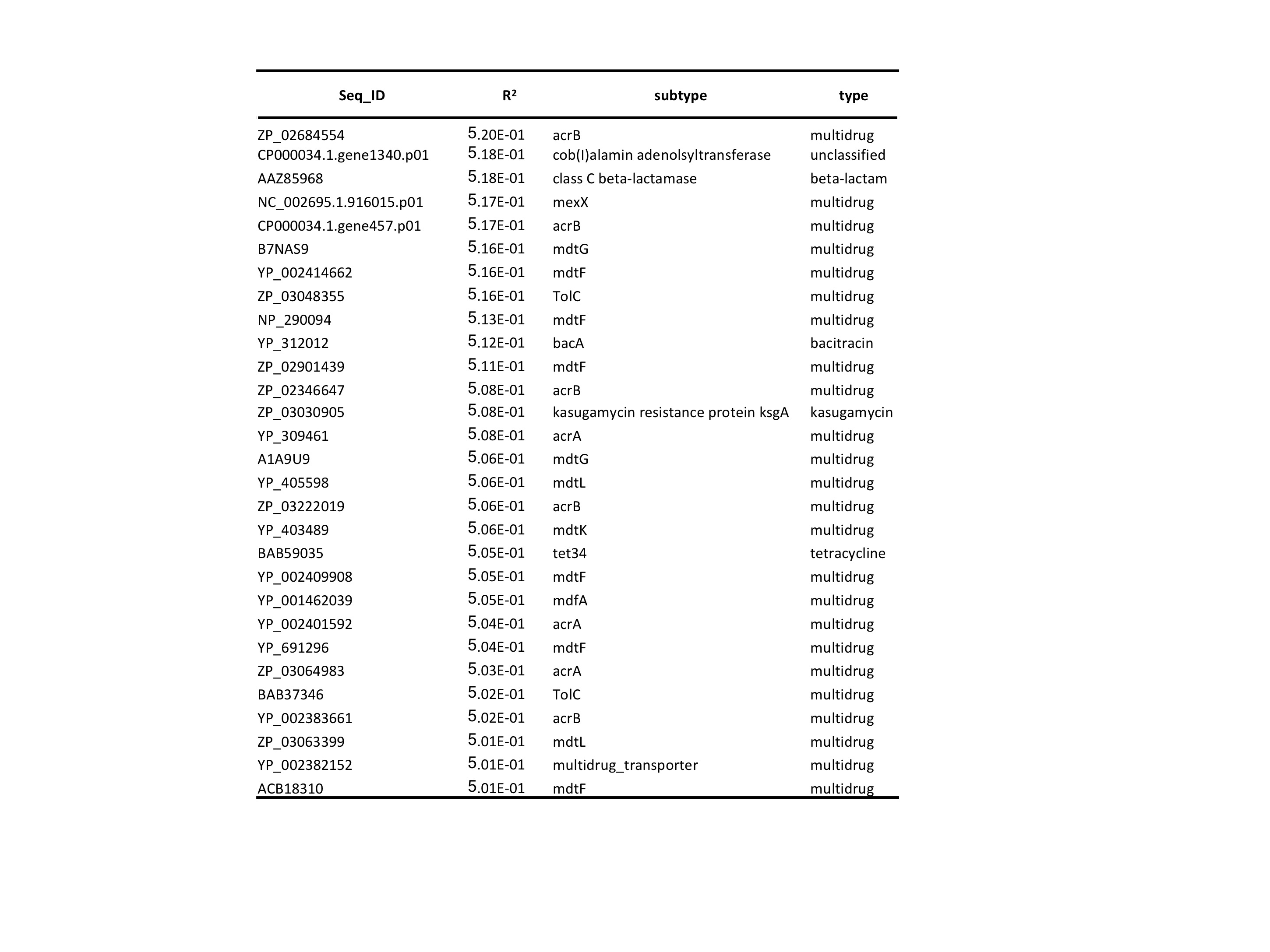
Table S7.** Sequence information of ARGs of relative high correlation (R^2^ ≥ 0.5) with overall abundance profiles.
